# Comparative Evaluation of Mechanical Properties and Color Stability of Dental Resin Composites for Chairside Provisional Restorations

**DOI:** 10.3390/polym16142089

**Published:** 2024-07-22

**Authors:** Haikun Yu, Jiaqi Yao, Zhili Du, Jingmei Guo, Wenlong Lei

**Affiliations:** 1 State Key Laboratory of Oral & Maxillofacial Reconstruction and Regeneration, Key Laboratory of Oral Biomedicine Ministry of Education, Hubei Key Laboratory of Stomatology, School & Hospital of Stomatology, Wuhan University, Wuhan 430072, China; 2 Department of Engineering Mechanics, School of Civil Engineering, Wuhan University, Wuhan 430072, China

**Keywords:** color stability, composite resins, mechanical tests, optical transparency, surface roughness, temporary dental restorations

## Abstract

Resin composites have become the preferred choice for chairside provisional dental restorations. However, these materials may undergo discoloration, changes in surface roughness, and mechanical properties with aging in the oral cavity, compromising the aesthetics, functionality, and success of dental restorations. To investigate the color and mechanical stability of chairside provisional composite resins, this study evaluated the optical, surface, and mechanical properties of four temporary restoration resin materials before and after aging, stimulated by thermal cycling in double-distilled water. Measurements, including CIE LAB color analysis, three-point bending test, nanoindentation, scanning electron microscopy (SEM), and atomic force microscopy (AFM), were conducted (n = 15). Results showed significant differences among the materials in terms of optical, surface, and mechanical properties. Revotek LC (urethane dimethacrylate) demonstrated excellent color stability (ΔE_00_ = 0.53-Black/0.32-White), while Artificial Teeth Resin (polymethyl methacrylate) exhibited increased mechanical strength with aging (*p* < 0.05, FS = 68.40 MPa-non aging/87.21 MPa-aging). Structur 2 SC (Bis-acrylic) and Luxatemp automix plus (methyl methacrylate bis-acrylate) demonstrated moderate stability in optical and mechanical properties (Structur 2 SC: ΔE_00_ = 1.97-Black/1.38-White FS = 63.20 MPa-non aging/50.07 MPa-aging) (Luxatemp automix plus: ΔE_00_ = 2.49-Black/1.77-White FS = 87.72 MPa-non aging/83.93 MPa-aging). These results provide important practical guidance for clinical practitioners, as well as significant theoretical and experimental bases for the selection of restorative composite resins.

## 1. Introduction

Dental resin composites have revolutionized dental care, enabling minimally invasive dentistry to preserve healthy tooth structure and provide natural-appearing esthetic prostheses. Due to their convenience of handling, excellent physicochemical properties, wide range of available colors, good biocompatibility, and ability to restore the natural appearance of teeth, resin-based composites have become the preferred choice for provisional restoration materials [1]. These provisional restorations play important roles, such as acting as thermal insulators, protecting the prepared tooth structure, stabilizing occlusion, and maintaining aesthetics [2]. However, materials degrade in the oral cavity over time, which could compromise aesthetics and functionality, leading to complications such as patient dissatisfaction and the need for replacement of the restoration. Therefore, materials used for provisional restorations should possess sufficient physical, chemical, and aesthetic properties and stability to ensure favorable aesthetics, functionality, and the success of the final restorations [3].

With advancements in technology, various resin materials and preparation methods have emerged clinically. Based on the composition, provisional restoration materials can be broadly divided into two main types [4]: (a) polymethyl methacrylate (PMMA) or polyethyl methacrylate (PEMA)-based and (b) bis-acrylic or dimethacrylates resins. Additionally, there are several other kinds of materials, such as epimine resin or polycarbonate. For several decades, PMMA has been one of the most commonly used provisional restorative materials [5]. Despite the advantages of convenience and low cost, PMMA has limitations, such as low color stability and mechanical properties depending on processing conditions and precision, leading to potential voids in the restoration and decreased performance [6]. Unlike PMMA, methyl methacrylate diethylene glycol dimethacrylate (bis-acrylic) composite resins contain monomers and filler particles, reducing polymerization shrinkage and heat generation and improving color stability [7]. Additionally, the use of automatic mixing syringes for bis-acrylic composite resins, though increasing costs, reduces performance degradation due to uncontrolled manual processing conditions. To improve the mechanical strength, wear resistance, and color stability of resin materials, different polymerization methods, filler compositions, and monomer types have been fabricated to ensure the desirable restoration outcomes, such as methyl methacrylate, bisphenol A glycerolate dimethacrylate (Bis-GMA), and urethane dimethacrylate (UDMA) [8].

After being placed in the oral cavity, restoration materials undergo an aging process due to factors like exposure to oral fluids, temperature fluctuation, and chewing wear. This can lead to a decrease in the marginal integrity of the restoration, resulting in microleakage and potentially causing complications such as dental caries and pulpitis [9]. The aging of these materials is a degradation process influenced by both their chemical composition and mechanical behavior. Additionally, the intraoral retention of resin composites is directly related to the material’s performance threshold, such as its flexural strength under pressure in the oral environment [10].

Changes in color, surface roughness, and other factors can also affect treatment outcomes. Discoloration, particularly in anterior teeth with high aesthetic requirements, significantly impacts the clinical longevity of restorations [3]. Given the variety of temporary restoration resin materials used in clinical practice, studying the aging behavior of these materials and comparing the changes in their optical, mechanical, and surface properties before and after aging is of significant value for selecting appropriate clinical restorative composite resins.

Therefore, this study aims to evaluate the changes in optical, surface, and mechanical properties of various chairside composite resins before and after simulating intraoral environments. This will provide theoretical and experimental bases for the application and optimization of temporary restorative resin materials. Additionally, understanding the underlying causes of performance variations may offer valuable insights for improving the material’s properties.

## 2. Materials and Methods

### 2.1. Materials

**Figure 1:**
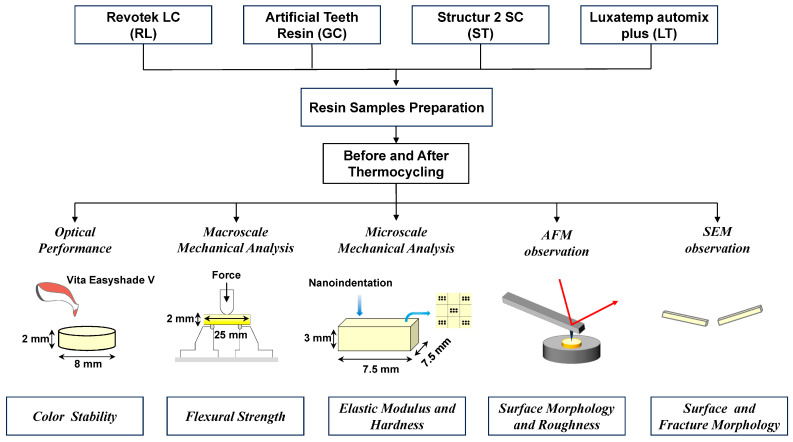
The diagram of the test design used in this study.

Considering factors such as the frequency of use, the properties of materials, and curing methods in clinical practice for temporary restorations, four commonly used chairside dental temporary restorative composite resins (Table 1) were selected to evaluate the optical, mechanical, and surface stability. Figure 1 describes the study design and flow of the specimens through this study. To simulate the intraoral aging process of dental composite resins, a thermal cycling procedure was employed [11]. The resin materials used in this study are denoted by their respective abbreviations: GC: Artificial Teeth Resin (polymethyl methacrylate); ST: Structur 2 SC (Bis-acrylic); LT: Luxatemp automix plus (methyl methacrylate bis-acrylate); and RL: Revotek LC (urethane dimethacrylate).

### 2.2. Sample Preparation

Composite resin samples were prepared in silicone molds according to the manufacturer’s instructions and covered with a polyester film and a glass slide for 10 min. For the light cure composites, samples were irradiated for 20 s with an LED light (Ivoclar Vivadent Bluephase G4 LED Curing Light). Surfaces were then washed with 70% ethanol, followed by ultrasonic cleaning in distilled water for 30 min. All samples were prepared by one operator and underwent the same procedures of grinding and polishing. Grinding was performed using silicon carbide sandpaper (grit sizes of 800, 1200, 2500, and 5000 sequentially), followed by polishing with diamond suspension (particle sizes of 3 and 1 μm). A digital caliper with a precision of 0.01 mm was used to ensure consistency in sample dimensions. The sample size was calculated by G-Power3.1(α = 0.05) and adjusted based on the results of a pilot study.

### 2.3. Aging Procedure

After the prepared samples were labeled and grouped randomly, specimens for the aging subgroups were placed in the double-distilled water bath of a thermal cycling machine (THE 1400, SD Mechatronik, Feldkirchen-Westerham, Germany), and the thermal cycles were conducted at temperatures from 5 to 55 °C with a 30 s dwell time in each bath for 5000 cycles [12]. After the water bath aging process, the samples were collected, dried, and kept in the dark.

### 2.4. Optical Analysis

To assess the color stability and transparency before and after aging, composite resin samples were prepared with circular silicone molds (diameter 8 mm, depth 2 mm) following the manufacturer’s guidelines. The surfaces of the samples (n = 15) were ground, polished, and then washed with 70% ethanol, cleaned in distilled water, and dried at 37 °C. Color measurements were then conducted using a spectrophotometer (VITA Easyshade V, Germany), according to the International Commission on Illumination (CIE) system [13]. Measurements were made against a white background (PVC plastic sheet) (L* = 93.3; a* = 1.2; b* = 5.4). Color differences (∆E_00_) were calculated using the CIEDE2000 Equation (1):

(1) $$\Delta E00=\sqrt{{\left(\frac{\Delta L’}{k_{L}S_{L}}\right)}^{2}+{\left(\frac{\Delta C’}{k_{C}S_{C}}\right)}^{2}+{\left(\frac{\Delta H’}{k_{H}S_{H}}\right)}^{2}+R_{T}\frac{\Delta C’}{k_{C}S_{C}}\frac{\Delta H’}{k_{H}S_{H}}}$$

Here, ∆L’, ∆C’, and ∆H’ represent the differences in luminance, chroma, and hue between the two color readings, respectively. The R_T_ function addresses the interaction between chroma and hue changes, especially in the blue region. S_L_, S_C_, and S_H_ are weighting functions used to adjust the perceived magnitude of color differences based on the position of the color coordinate differences. Additionally, k_L_, k_C_, and k_H_ serve as correction factors under experimental conditions [14].

Measurements were also made against a black background (PVC plastic sheet) (L* = 27.9; a* = 0.0; b* = 0.0). Transparency (TP) was calculated using the following Equation (2), where W and B represent the white and black backgrounds, respectively.

(2) $$TP=\sqrt{{\left({L_{w}}^{*}-{L_{b}}^{*}\right)}^{2}+{\left({a_{w}}^{*}-{a_{b}}^{*}\right)}^{2}+{\left({b_{w}}^{*}-{b_{b}}^{*}\right)}^{2}}$$

### 2.5. Macroscale Mechanical Analysis

According to ISO standards [15], two batches of samples (n = 15) were prepared with silicone molds of dimensions 25 mm × 2 mm × 2 mm. With or without aging treatment, the samples were ground, polished, and then washed with 70% ethanol, cleaned in distilled water, and air-dried. Samples were placed on a custom fixture with two supports spaced 20 mm apart and mounted on a universal testing machine (ElectroForce 3220, TA Instruments, New Castle, DE, USA). A load was then applied at the center of the sample and steadily increased at a crosshead speed of 5 mm/min until the sample broke. The real-time radial displacement–stress graph was the output, with the inflection point of linearity loss considered the breaking point. The flexural strength (FS) was calculated using the following Equation (3).

(3) $$FS(\mathrm{MPa})=3FL/2bt^{2}$$

where F is the maximum force applied during the bending test, L is the distance between supports, b is the width of the sample, and t is the thickness of the sample.

### 2.6. Microscale Mechanical Analysis

The composite resin samples were prepared with the silicone molds of 7.5 mm × 7.5 mm × 3 mm and cured according to the manufacturer’s instructions. Samples of each material were tested both before and after aging (n = 4). One batch of samples was encapsulated in epoxy resin, then ground and polished to expose a smooth, flat surface for testing. Another batch underwent thermal cycling in a water bath, followed by rinsing with 70% ethanol, cleaning with distilled water, and air drying. Then, these samples were encapsulated in epoxy resin and subjected to the same grinding and polishing process. Grinding was performed using silicon carbide sandpaper (grit sizes of 800, 1200, 2500, and 5000 sequentially), followed by polishing with diamond suspension (particle sizes of 3 and 1 μm) [16].

Nanoindentation tests were conducted using a commercial TriboIndenter system (Hysitron TI 980, Bruker, Billerica, MA, USA) with a conical diamond indenter. The indenter had a tip radius of curvature and cone angle of 5 μm and 60 μm, respectively. Indentation tests were performed in load-controlled mode, where a programmed load function controlled the applied load, and displacement was continuously monitored by the computer. The load function included 5 s of linear loading, 5 s of unloading, and a 5 s hold at a peak load of 3000 μN to minimize creep. Hardness and elastic modulus were determined from the load–displacement curves, following the method reported in a previous study [17]. The values for hardness and elastic modulus were obtained from the average results at six indentation points at different locations. The spacing between indentation points was set to 100 μm to eliminate interactions between adjacent indents.

### 2.7. Atomic Force Microscopy (AFM) Characterization

AFM measurements were carried out to evaluate the composite resin surfaces at regular temperature using Atomic Force Microscope (MultiMode 8-HR, Bruker, Billerica, MA, USA). Experiments were conducted over scan areas of 200 μm × 200 μm and 50 μm × 50 μm (both with a vertical range of 15 μm). All images were acquired in ScanAsyst^®^ mode with a resolution of 512 × 512 pixels. Images were then further processed and analyzed using NanoScope Analysis 1.8 software, assessing surface morphology and obtaining surface root mean square roughness (Rq), average roughness (Ra), and the surface three-dimensional reconstruction morphology.

### 2.8. Morphology Observation

Composite resin samples were encapsulated in epoxy resin and surface-polished and then examined for their surface morphology using scanning electron microscopy (Zeiss Sigma, Oberkochen, Germany). Observations were performed using a secondary electron detector at an acceleration voltage of 5 kV on gold-coated specimens. The composite resin surfaces of samples before and after aging were examined at various magnifications, and five images in each specimen were randomly captured. Additionally, the fractured surfaces from three-point bending tests were gold-coated and examined at different magnifications to compare the morphology of the surfaces before and after aging.

### 2.9. Statistical Analysis

After the normality and homoscedasticity of the data were confirmed beforehand, one-way ANOVA was used to determine the statistically significant differences of the materials on color difference (ΔE_00_) and transparency (TP) before and after aging procedure. Two-way ANOVA was conducted to analyze the effect of two variables (materials and thermocycling) on bending strength (FS), elastic modulus (Er), hardness (H), and roughness (Ra) with the Statistical Package of Social Sciences (SPSS v.23). The level of statistical significance for all analyses was set at 0.05 (α = 0.05). For morphologies scanned by SEM and AFM, representative areas were randomly selected and captured.

## 3. Results and Discussion

### 3.1. Optical Performance

**Figure 2:**
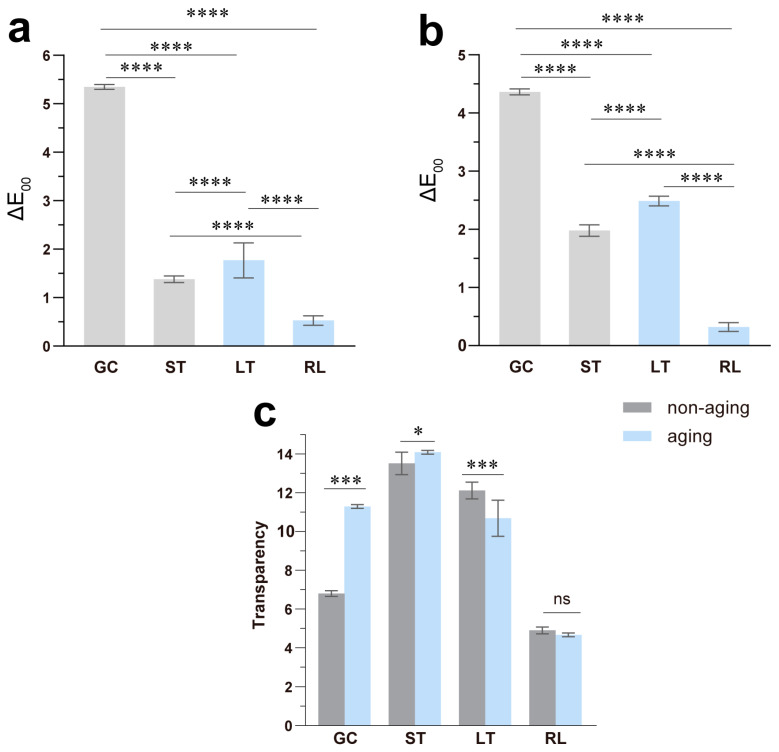
The optical performance of the composite resins. (**a**) ΔE_00_ on the white and (**b**) black background; (**c**) transparency differences of the composite resins before and after aging. * *p* < 0.05, *** *p* < 0.001, **** *p* < 0.0001; ns, not significant.

The color and translucency of dental composite resins can be influenced by various factors, including the resin’s intrinsic matrix and fillers, polymerization process and conditions, surface treatments and polishing procedures, as well as aging and intraoral environmental factors [18]. The results of the optical performance before and after aging are shown in Figure 2, which summarizes the color difference (ΔE_00_) and transparency (TP) data. Detailed numerical values of the CIE color system are presented in Table 2 and Table 3. The color differences ΔE_00_ among the materials are ranked in descending order as GC, LT, ST, and RL. Notably, GC exhibits significantly higher color differences compared to the other resin materials against black and white backgrounds (*p* < 0.05), with ΔE_00_ > 2.25 on both backgrounds. This value surpasses the 50% acceptable threshold for dental material color difference [19]. Additionally, LT demonstrates ΔE_00_ > 2.25 against a black background. Conversely, RL and LT register lower ΔE_00_ values against both backgrounds, with ΔE_00_ < 2.25.

Regarding transparency differences, RL and LT exhibit a decrease in transparency after aging, while ST and GC demonstrate a significant increase (*p* < 0.05). Notably, GC’s variation in transparency is significant (*p* < 0.001), while the composite variance of RL pre- and post-aging is comparatively minimal (*p* > 0.05). Furthermore, the transparency of ST and LT, both dispensed from automatic mixing syringes, surpasses that of GC and RL. It is well known that composite resins are semi-transparent materials composed of monomers with high transparency, nano- and micrometer-sized filler particles, and various mixing components such as photosensitive and thermosensitive initiators. When light passes through the composite resin, it is absorbed and scattered by the filler particles and mixing components before reaching the observer’s eye. The transmitted light through the material serves as the basis and energy source for initiating photosensitive reactions, thereby promoting polymerization and monomer conversion [20]. The literature suggests that composite resins have higher transparency when the refractive index of the base monomer is similar to the filler’s. Firstly, it reduces light reflection and scattering: When the refractive index of the monomer in composite resins matches that of the filler, there is a reduction in light reflection and scattering at the interface between them. This minimizes energy loss and allows light to pass through the material more easily, thereby increasing transparency. Secondly, it reduces optical non-uniformity: Refractive index matching helps achieve this by ensuring more uniform refraction and transmission of light within the material. This, in turn, decreases scattering and chromatic dispersion, further enhancing the material’s transparency. Lastly, it enhances light transmission efficiency: Matching refractive indexes improves the efficiency of light transmission within the material, allowing light to pass through more effectively without excessive reflection or scattering at the interfaces [21]. Furthermore, the transparency of composite resins is influenced by both the wt % of the fillers and the similarity between the filler particle size and the wavelength of incident light. As a result, both internal agglomeration and external lighting conditions affect the optical properties of composite materials [22].

Therefore, the observed differences in the transparency of the four composite resins before and after aging may be caused by the changes in their internal microstructure network due to thermal cycling. These changes could potentially alter the refractive index and consequently affect transparency. In summary, based on the optical performance evaluation, RL exhibits the most optimal color stability, whereas GC displays inferior color stability. ST and LT, dispensed from automatic mixing syringes, occupy an intermediary position. Therefore, clinically, RL might be preferable for patients seeking optimal aesthetics in temporary restorations, particularly in the anterior region.

### 3.2. Flexural Strength

**Figure 3:**
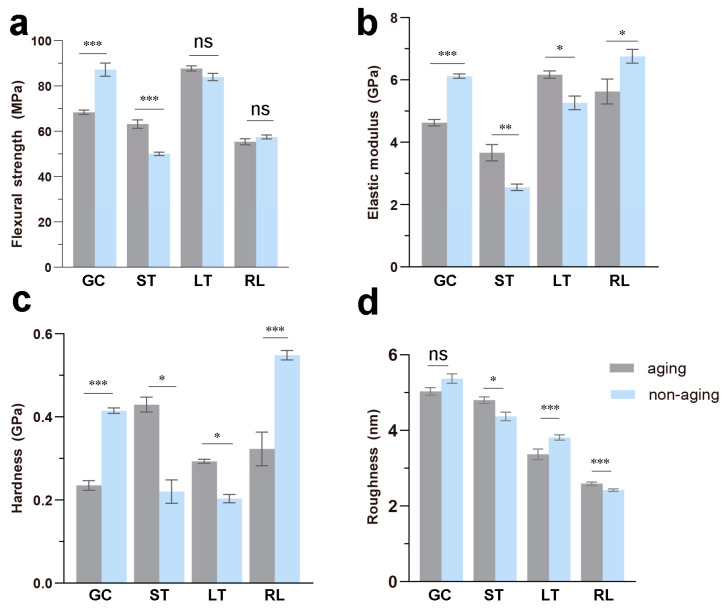
Mechanical properties and roughness of the composite resins. (**a**) Flexural strength, (**b**) elastic modulus, (**c**) hardness, and (**d**) surface roughness of the composite resins before and after aging. * *p* < 0.05, ** *p* < 0.01, *** *p* < 0.001; ns, not significant.

The macroscopic mechanical properties of different composite resins are represented in Figure 3. Flexural strength is a crucial factor influencing the strength of composite resins. Figure 3a illustrates that the flexural strength of the composites before aging obtained in the present study is in the range of 55 to 90 MPa, and these results are consistent with those reported in the literature [4]. After three-point bending tests, the bars were fractured into two sections of nearly equal length, indicating uniformity within the composite resins without significant stress concentration points. Clear differences emerged in flexural strength after aging. GC exhibited the highest flexural strength, followed by LT, RL, and ST. Notably, GC demonstrated a significant increase in flexural strength after aging (*p* < 0.001), while ST showed a decrease (*p* < 0.001), and the changes in LT and RL were relatively small (*p* > 0.05).

With aging in the oral cavity, dental composites may undergo processes like resin component leaching, accelerated water absorption and dissolution, swelling, and resin matrix degradation, ultimately affecting their performance [23]. By simulating the oral aging processes through thermal cycling, this study mimicked composite resin degradation. Here, LT and RL exhibited relatively better stability in flexural strength.

### 3.3. Elastic Modulus and Hardness

The elastic modulus and hardness of the four composite resins before and after aging are shown in Figure 3b,c. Both the elastic modulus and hardness are physical quantities representing the material’s resistance to deformation. The trends observed for both parameters were similar and closely related to flexural strength. After aging, the elastic modulus and hardness of GC and RL increased (*p* < 0.001, *p* < 0.05), while those of ST and LT decreased (*p* < 0.01, *p* < 0.05). It is noteworthy that apart from GC, the data dispersion of the elastic modulus and hardness for the three light-cured composite resins (ST, LT, RL) is relatively large. Further analysis of the original data revealed a difference in the mechanical performance between data points from the periphery of the sample block and those from the interior. This suggests variations in mechanical properties depending on the depth of light curing within the light-cured composite resins [24]. This finding provides valuable guidance for clinical use: comprehensive light curing of temporary restorations should be prioritized as much as possible to minimize performance discrepancies within them, improving the retention time of the composite resins to ensure treatment effectiveness [25].

### 3.4. Surface Roughness

The surface roughness of the four resin materials before and after aging is depicted in Figure 3d. Notably, the ranking of surface roughness remained unchanged before and after aging, with GC having the highest roughness, followed by ST, LT, and RL. After aging, LT exhibited an increase in surface roughness (*p* < 0.001), while ST (*p* < 0.05) and RL (*p* < 0.001) showed a decrease. Interestingly, GC, which had the highest roughness before aging, displayed an increase in surface roughness after aging as well, although this difference was not statistically significant (*p* > 0.05). The observed changes in surface roughness of GC and LT are consistent with their respective changes in color difference (ΔE_00_). This phenomenon can be explained by the interaction of light with the composite resins’ surfaces. A smooth surface reflects light more predictably to the observer’s eye, resulting in a more stable perceived color. Conversely, a rough surface scatters light more unpredictably, potentially leading to a greater perceived difference in color [26,27].

**Figure 4:**
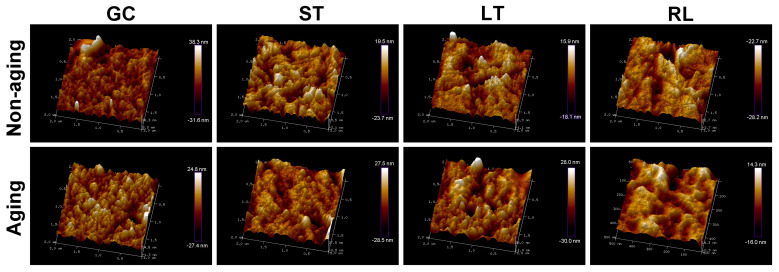
Representative regions of the composite resins observed by AFM.

**Figure 5:**
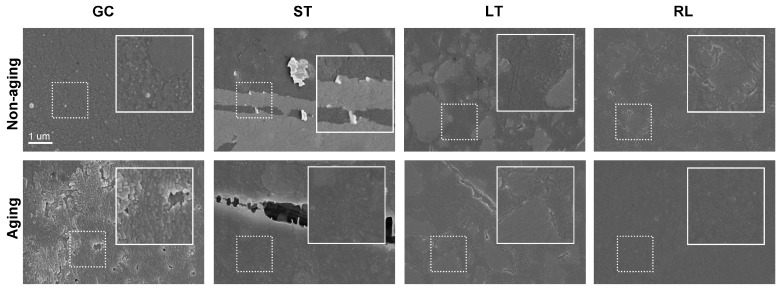
The representative regions of the surface morphology of the composite resins.

The surface topography of the tested composites is shown in Figure 4 and Figure 5. The significant difference in surface roughness and optical stability between RL and GC might be attributed to their material compositions. GC is a traditional powder-liquid mixed material, while RL is the only pre-polymerized material. This pre-polymerization process in RL could potentially contribute to a higher degree of polymerization and lower residual monomer content compared to GC [28,29]. Due to the inherent uncertainties associated with manual mixing, GC might have a lower polymerization rate and, consequently, larger residual monomer particles. This could also explain why ST and LT, dispensed from auto-mixing injector systems, exhibit better optical stability compared to GC but are inferior to RL in this aspect. However, further testing to quantify resin conversion rates would be necessary to definitively support this hypothesis.

### 3.5. Surface Morphology

Scanning electron microscopy (SEM) images in Figure 5 further confirm the surface roughness findings. GC showed a consistently rougher surface morphology before and after aging, which aligns with its greater ΔE_00_ values. Conversely, RL, characterized by lower surface roughness and smaller ΔE_00_, exhibited a smoother surface texture in the SEM scans. For clinical application, the surface of the restoration should be as smooth as possible to ensure the aesthetic properties of the restoration, including color stability and transparency [30]. Thorough polishing can be a valuable tool to optimize the surface roughness of restorative materials. This not only enhances aesthetics but also reduces plaque accumulation, thereby lowering the risk of secondary caries and improving the intraoral retention of restorations, preventing potential dislodgement [31].

### 3.6. Fracture Morphology Analysis

**Figure 6:**
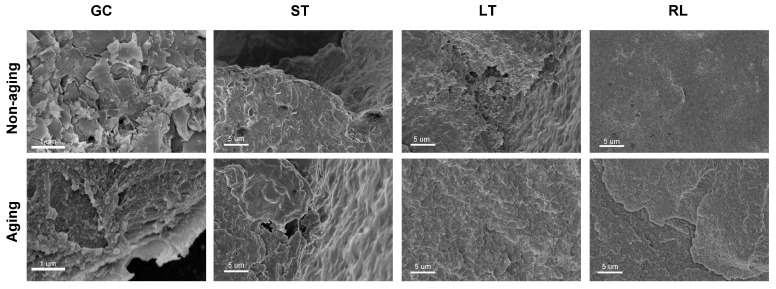
The representative SEM images of fracture surface of the composite resins before and after aging via three-point bending.

Representative SEM images of fractured specimens are shown in Figure 6. The cross-section of the powder–liquid mixed resin GC before aging reveals the presence of incompletely polymerized solid particles distributed on the fractured surfaces. These unreacted particles hinder homogeneous integration, manifesting as hilly features, sharp, thin flakes, and clustered protrusions. This disrupts the density of the composites’ internal network and compromises the uniformity of stress transmission, ultimately leading to relatively small values in macroscopic flexural strength [32,33,34].

Interestingly, the cross-section of GC becomes denser and more uniform after aging, which aligns with the observed increase in its flexural strength. We propose a potential explanation for this observation. During sample preparation, the center of the composite resin samples is inherently more challenging to fully polymerize compared to the surface regions. Furthermore, GC, being the only powder-liquid mixed composite resins, may have a lower intrinsic crosslinking density due to manual preparation, especially near the center of the samples [33]. The thermal cycling process likely introduces additional energy through temperature fluctuations. This might have facilitated further polymerization and crosslinking of the incompletely polymerized regions within GC, particularly those near the center. The previously dispersed solid particles could then integrate more effectively into the resin network. These solid particle protrusions, which initially compromised mechanical strength, might now become reinforcing elements, contributing to the significant increase in macroscopic mechanical strength observed after aging.

In addition, the increase in GC flexural strength may also be related to stress relief and structural optimization. Previous studies [18,35,36,37] have indicated that resin materials may develop internal stresses during curing, which can weaken their mechanical strength. Environmental factors such as thermal cycling or water absorption during aging can help relieve these internal stresses. Temperature changes lead to a gradual reduction of internal stresses, facilitating a more uniform distribution of stress within the material. At the same time, polymer chains in PMMA can relax and reorganize under thermal stress. This relaxation process involves adjustments in molecular chains to minimize internal stresses, thereby enhancing the material’s mechanical properties, such as flexural strength. As stress relaxation progresses, PMMA may exhibit increased toughness and resistance to crack propagation, characteristics that can be observed in flexural strength tests following thermal cycling [38,39]. For ST, the observed differences before and after aging can be attributed to the appearance of solid filler particle extrusion after aging. This phenomenon results in clearly visible extrusion holes and gaps on the cross-section, contributing to a decrease in flexural strength.

This study evaluated the properties of four commonly used provisional restorative resin materials. While there is currently no unified standard to definitively determine the “superior” or “inferior” properties of dental composite resins [6], the results of this study from color measurement, mechanical analysis, and two- and three-dimensional morphology observations revealed that the properties before and after aging are significantly influenced by the type of material, leading us to reject the null hypothesis.

As previously mentioned, there has been limited research on the four types of provisional restorative composite resins used in this study. The available previous studies generally align with the conclusions of this study [40,41,42,43]. The dental temporary restorative resin materials exhibit distinct characteristics in terms of optical, macroscopic, and microscopic mechanical properties. When placing temporary restorations in the posterior region, where high mechanical strength is crucial, or for patients who frequently consume hot beverages, GC might be a suitable choice due to its potential for increased mechanical strength over time. Conversely, if the restoration requires a shorter placement duration or is intended for the aesthetically critical anterior region, prioritizing RL might be advisable due to its stable mechanical strength and superior color stability. However, in the selection of provisional restorative composite resins, in addition to considering the aforementioned aspects, factors such as the thickness, the coverage area, the clinician’s skill level, the treatment conditions of the dental office, and the patient’s oral condition and habits should also be taken into consideration.

## 4. Conclusions

This study evaluated four provisional restorative composite resins, revealing significant differences influenced by material type and aging. The results indicated that GC exhibits increased mechanical strength after aging, making it suitable for posterior restorations requiring high durability, while RL offers superior color stability, which is ideal for anterior restorations. These findings may provide valuable insights for optimizing provisional restorations in clinical practice.

## Figures and Tables

**Table 1:** The composite resins used in this study.

Materials	Manufacture	Main Components *	Application Modes
Revotek LC (RL)	GC Dental Products Co., Tokyo, Japan	Urethane dimethacrylate (UDMA), trimethacrylate, silicon dioxide, amorphous, butylated hydroxytoluene (BHT), iron (III) oxide, and titanium dioxide	Light-cured single-component sculptable composite resin
Artificial Teeth Resin (GC)	New Century Dental, Shanghai, China	Polymethyl methacrylate (PMMA) mold powder, benzoyl peroxide (BPO), and trace pigments	Mold powder used in combination with denture base resin-type liquid and heat-cured
Structur 2 SC (ST)	VOCO, GmbH, Hanau, Germany	Methacrylates, amines, terpenes, benzoyl-peroxide, and butylated hydroxytoluene	A fluorescent cold polymerizing paste–paste system that consists of base paste and catalyst paste molded by syringe
Luxatemp Automix Plus (LT)	DMG, Hamburg, Germany	Barium glass, bisphenol A bismethacrylate, carbamate dimethacrylate, triethylene glycol dimethacrylate, camphor quinone, and pigments	As a self-curing composite resin, the matrix and catalyst are mixed with a syringe and molded directly

* Information was provided by the manufacturers.

**Table 2:** The mean CIE LAB color coordinates ± SD (n = 15, white background).

Material	Aging	CIE LAB Color Coordinates		
		L	a	b
GC	Before	84.37 ± 0.11	2.43 ± 0.10	31.53 ± 0.15
	After	83.60 ± 0.10	2.34 ± 0.05	31.50 ± 0.08
LT	Before	80.85 ± 0.08	0.82 ± 0.23	28.75 ± 0.50
	After	80.95 ± 1.42	1.58 ± 0.05	32.05 ± 0.07
ST	Before	85.52 ± 0.08	−0.61 ± 0.03	27.50 ± 0.10
	After	86.33 ± 0.11	−0.27 ± 0.04	30.33 ± 0.08
RL	Before	87.57 ± 0.10	1.68 ± 0.08	36.70 ± 0.08
	After	85.81 ± 0.08	2.35 ± 0.07	52.38 ± 0.08

**Table 3:** The mean CIE LAB color coordinates ± SD (n = 15, black background).

Material	Aging	CIE LAB Color Coordinates		
		L	a	b
GC	Before	81.29 ± 0.11	1.02 ± 0.04	28.04 ± 0.20
	After	80.87 ± 0.07	0.91 ± 0.05	28.02 ± 0.09
LT	Before	72.47 ± 0.13	−1.81 ± 0.05	20.45 ± 0.05
	After	73.76 ± 0.04	−1.01 ± 0.11	24.65 ± 0.09
SC	Before	74.29 ± 0.07	−3.22 ± 0.06	18.38 ± 0.08
	After	75.98 ± 0.08	−3.09 ± 0.09	21.20 ± 0.09
RL	Before	83.35 ± 0.05	1.68 ± 0.07	31.37 ± 0.10
	After	81.33 ± 0.08	−0.41 ± 0.06	42.39 ± 0.08

## Data Availability

The original contributions presented in the study are included in the article, further inquiries can be directed to the corresponding author.
